# Sudden, Severe, Refractory Hypotension Following Sugammadex Administration During Low‐Risk Orthopedic Surgery in a Healthy Young Adult: A Case Report

**DOI:** 10.1155/cria/4061273

**Published:** 2026-09-20

**Authors:** Bibek Devkota, Marcus Wilson, Babita Bhattarai

**Affiliations:** ^1^ Department of Anesthesiology, University of Wisconsin Hospital and Clinics, Madison, Wisconsin, USA, uwhealth.org; ^2^ Department of Medicine, Nepalese Army Institute of Health Sciences, Kathmandu, Nepal, naihs.edu.np

**Keywords:** anaphylaxis, hypersensitivity, hypotension, neuromuscular blockade reversal, sugammadex

## Abstract

Sugammadex is widely used for rapid and predictable reversal of aminosteroid neuromuscular blockade and has a favorable safety profile. However, rare cardiovascular adverse events, including severe hypotension, bradycardia, and asystole, have been reported. We describe a case of a 39‐year‐old healthy male who developed profound hypotension without bradycardia approximately six minutes after receiving sugammadex for reversal of moderate neuromuscular blockade following uncomplicated orthopedic surgery. His blood pressure decreased from 95/51 mmHg at baseline to 68/33 mmHg six minutes after sugammadex administration, reaching a nadir of 50/29 mmHg at 17 min, with a heart rate of 117 beats/min. The patient had stable intraoperative hemodynamics and no evidence of bronchospasm, cutaneous manifestations, or airway compromise at the time of the event. Hypotension was refractory to repeated boluses of phenylephrine and ephedrine, with subsequent improvement following norepinephrine administration. The patient recovered without further complications and was discharged the same day. Although causality cannot be definitively established, the temporal relationship raises concern for sugammadex‐associated hypotension without bradycardia, possibly related to an atypical hypersensitivity reaction. This case highlights that severe hypotension may occur even in low‐risk patients in the absence of bradycardia or other classic features of anaphylaxis, requiring prompt recognition and early potent vasopressor support.

## 1. Introduction

Sugammadex is a modified γ‐cyclodextrin designed for the rapid reversal of aminosteroid neuromuscular blocking agents most notably for rocuronium and vecuronium [1]. Since its approval in 2015, it has increasingly replaced traditional anticholinesterase‐based reversal strategies due to its rapid, predictable, and cholinergic side‐effect–free mechanism of action. Compared with neostigmine–glycopyrrolate combinations, sugammadex provides faster reversal, higher train‐of‐four (TOF) ratios at extubation, and a reduced risk of postoperative residual neuromuscular blockade, thereby improving perioperative safety and recovery profiles [2, 3]. Despite its favorable safety profile, sugammadex is associated with recognized adverse effects and carries specific warnings and precautions in its FDA‐approved prescribing information. These include hypersensitivity and anaphylactic reactions, bradycardia, and other cardiovascular effects [4]. Cases of severe cardiovascular instability, including profound hypotension, marked bradycardia, and, rarely, cardiac arrest or asystole, have also been reported in the literature [5–7]. The exact mechanisms remain incompletely understood, but proposed mechanisms may involve hypersensitivity reactions, direct hemodynamic effects, or autonomic imbalance following rapid neuromuscular recovery [8]. Although large studies suggest a lower incidence of perioperative complications with sugammadex compared to traditional neostigmine–glycopyrrolate combination, few published case reports of adverse cardiovascular events highlight the need for heightened vigilance, especially in high‐risk patients [6, 9–11]. Here, we present a case of sugammadex‐associated severe refractory hypotension in an otherwise healthy patient, aiming to emphasize its clinical presentation, potential mechanisms, and implications for perioperative management.

## 2. Case Report

A 39‐year‐old physically active male (BMI 19 kg/m^2^) presented with a displaced fracture of the lateral end of the clavicle following a contact sports injury. His medical history included well‐controlled hypertension managed with lifestyle modification and gastroesophageal reflux disease treated with as‐needed famotidine. He had previously undergone adenoidectomy, tonsillectomy, and inguinal hernia repair without anesthesia‐related complications and reported an allergy to mefloquine. Preoperative evaluation revealed stable vital signs (blood pressure 117/66, pulse rate 73), excellent functional capacity (> 10 METs), and a predicted uncomplicated airway. General anesthesia with endotracheal intubation was planned, followed by a postoperative interscalene brachial plexus block for analgesia. Anesthesia was induced with fentanyl 50 mcg, propofol 200 mg, lidocaine 100 mg, and rocuronium 70 mg, and the trachea was intubated uneventfully using video laryngoscopy (McGrath). Anesthesia was maintained with sevoflurane, with additional fentanyl at incision, dexamethasone, and hydromorphone given toward the end of surgery. Intraoperative hemodynamics remained stable with occasional phenylephrine boluses.

At the end of the procedure, the patient’s vital signs were stable with a blood pressure of 95/51 mmHg, heart rate 69 beats/min, and oxygen saturation 98% while on pressure support ventilation. Neuromuscular monitoring showed a TOF count of 2/4, indicating moderate blockade, and sugammadex 200 mg (∼2.8 mg/kg) was administered. Within 2 min, TOF recovered to 4/4 with a T4/T1 ratio of 105%. The patient remained hemodynamically stable on pressure support ventilation while we awaited further emergence from anesthesia.

About 6 min after sugammadex, the patient suddenly became hypotensive (BP 68/33 mmHg, HR in the 90s) with oxygen saturation 99%. A 100‐mcg phenylephrine bolus was given, but hypotension persisted (72/35 mmHg), prompting a second bolus. A contralateral blood pressure check was performed—though this was a healthy young adult; hypotension after sugammadex was unexpected. Anaphylaxis was considered less likely given the absence of typical clinical features, including cutaneous manifestations, facial swelling, bronchospasm, increased airway pressures, or impaired oxygenation. Intravenous fluid boluses, additional phenylephrine, and ephedrine were administered over the next 10 min with minimal hemodynamic response. Ultimately, the patient responded to two boluses of norepinephrine (32 mcg total), after which blood pressure improved. As a precautionary measure for possible atypical hypersensitivity reaction, famotidine and diphenhydramine were administered. After hemodynamic stabilization, the patient was extubated in the operating room and observed briefly before transferring to the postanesthesia care unit (PACU). In PACU, vital signs remained stable, and the patient was awake, following commands, and maintaining oxygenation on room air without recurrence of hypotension. Given the intraoperative event, the planned interscalene brachial plexus block was deferred after discussion with the patient. He was discharged home later the same day in a stable condition. The timeline of the patient’s hemodynamic changes and pharmacologic interventions following sugammadex administration is summarized in Table 1.

**TABLE 1:** Timeline of hemodynamic events and pharmacologic interventions postsugammadex reversal of neuromuscular blockade.

Time after sugammadex administration (in minutes)	Noninvasive blood pressure (mmHg)	Heart rate (beats/min)	Pharmacological/other significant intervention
Before induction of anesthesia	117/66	73	Continued monitoring
0	95/51	69	Sugammadex 200 mg (∼2.8 mg/kg) administered IV; baseline monitoring
2	92/52	59	Continued monitoring; TOF ratio recovered to 105%
6	68/33	92	Hypotension noted; remeasured with secondary blood pressure cuff
7	72/35	94	Phenylephrine 100 mcg IV
8	68/32	97	Phenylephrine 100 mcg IV
9	61/34	98	Ephedrine 5 mg IV
11	58/32	118	Phenylephrine 200 mcg IV
12	53/27	116	Phenylephrine 200 mcg IV
14	55/33	107	Continued monitoring, IV fluid bolus ongoing
15	56/30	110	Continued monitoring
17	50/29	117	Norepinephrine 16 mcg IV
19	59/26	96	Norepinephrine 16 mcg IV
21	68/37	89	Diphenhydramine 12.5 mg IV, famotidine 20 mg IV
22	112/51	88	Continued monitoring
23	117/53	86	Continued monitoring
25	119/54	95	Transferred to PACU

*Note:* IV, intravenous; min, minutes; mg, milligram; mcg, microgram.

Abbreviations: PACU, Postanesthesia Care Unit; TOF, Train‐of‐four.

## 3. Discussion

Our case describes profound hypotension with marked tachycardia approximately 6 minutes after sugammadex administration in an otherwise healthy patient, without associated bradycardia, cutaneous manifestations, hypoxemia, bronchospasm, or airway pressure changes. The hypotension was poorly responsive to phenylephrine and ephedrine but improved after norepinephrine. This presentation differs from many previously reported severe cardiovascular events following sugammadex, which have included bradycardia, asystole, cardiovascular collapse, or accompanying features of hypersensitivity [6, 7, 9, 10, 12, 13].

Several reports more closely resemble the cardiovascular phenotype observed in our patient. Jeyadoss et al. described profound hypotension with tachycardia without initial cutaneous or airway manifestations; however, subsequent elevation of serum tryptase and positive intradermal testing supported sugammadex‐induced anaphylaxis [14]. Another report described initial hypotension and tachycardia without skin findings, followed by generalized erythema, periorbital edema, and respiratory manifestations [12]. Koo et al. reported severe hypotension (44/27 mmHg) with tachycardia (120 beats/min) approximately 2 min after sugammadex administration, without cutaneous or mucosal manifestations; however, the patient also developed hypoxemia, with an SpO_2_ of 93% despite 100% oxygen [15]. In contrast, our patient had isolated cardiovascular instability without subsequent cutaneous or respiratory manifestations.

The cardiovascular phenotype in our patient also provides insight into potential mechanisms. The absence of bradycardia and the compensatory increase in heart rate to approximately 117 beats/min make isolated vagally mediated cardiac depression less likely as the primary explanation. The limited response to phenylephrine and ephedrine followed by improvement with norepinephrine may be compatible with substantial reduction in vascular tone, although the absence of invasive hemodynamic measurements prevents definitive identification of reduced systemic vascular resistance, altered venous capacitance, or another mechanism. Proposed mechanisms of sugammadex‐associated cardiovascular instability include direct vasodilation, transient autonomic imbalance, changes in cardiac output, coronary vasospasm, and hypersensitivity reactions [8, 13, 16]. The heterogeneity of reported presentations suggests that more than one mechanism may contribute.

Hypersensitivity remains an important consideration because sugammadex‐associated anaphylaxis can present predominantly with cardiovascular compromise and does not invariably include early cutaneous or respiratory manifestations [17, 18]. However, our patient did not have peri‐event serum tryptase measurement or available allergy testing, and therefore an immunologic mechanism cannot be confirmed. The patient was referred for outpatient allergy evaluation, but results were not available at the time of manuscript preparation. A recent multicenter retrospective analysis of 30 patients with skin‐test–confirmed sugammadex hypersensitivity found hypotension to be the most common clinical manifestation, occurring in 83.3% of cases, followed by flushing or erythema in 70%; the median time to recognition was 5 min (IQR, 2–7.5 min) [19]. These findings support hypotension as a prominent manifestation of sugammadex hypersensitivity, while demonstrating that cutaneous manifestations are not universal.

Other reported causes of severe perioperative hypotension, including arrhythmia, hypovolemia, hemorrhage, excessive anesthetic effect, pulmonary embolism, and coronary vasospasm were less consistent with the clinical course and temporal relationship to sugammadex administration. In a pharmacovigilance analysis of the FDA Adverse Event Reporting System (FAERS), Mao et al. identified 1452 reports in which sugammadex was recorded as the primary suspect drug between January 2009 and September 2023. Hypotension was among the adverse events identified in postmarketing surveillance; however, spontaneous reporting systems cannot provide reliable incidence or prevalence estimates because the number of exposed patients and reporting rates are unknown [20, 21]. Nevertheless, these reports, together with the published case literature, support severe hemodynamic instability as a rare but recognized adverse event following sugammadex.

## 4. Conclusion

Sugammadex can rarely be associated with profound hypotension occurring without bradycardia or overt cutaneous or respiratory manifestations. The clinical presentation in this case is compatible with a predominantly vasodilatory or atypical hypersensitivity‐related response, although the absence of peri‐event tryptase and allergy testing precludes confirmation of the mechanism.

## Funding

No funding was received for this manuscript.

## Conflicts of Interest

The authors declare no conflicts of interest.

## Data Availability

The data that support the findings of this study are available on request from the corresponding author. The data are not publicly available due to privacy or ethical restrictions.
